# Green Silver Nanoparticles Promote Inflammation Shutdown in Human Leukemic Monocytes

**DOI:** 10.3390/ma15030775

**Published:** 2022-01-20

**Authors:** Mariafrancesca Cascione, Loris Rizzello, Daniela Manno, Antonio Serra, Valeria De Matteis

**Affiliations:** 1 Department of Mathematics and Physics “Ennio De Giorgi”, University of Salento, Via Arnesano, 73100 Lecce, Italy; mariafrancesca.cascione@unisalento.it (M.C.); daniela.manno@unisalento.it (D.M.); antonio.serra@unisalento.it (A.S.); 2 Department of Pharmaceutical Sciences (DISFARM), University of Milan, Via G. Balzaretti 9, 20133 Milan, Italy; loris.rizzello@unimi.it; 3 Institute for Bioengineering of Catalonia (IBEC), The Barcelona Institute of Science and Technology, Baldiri Reixac 10–12, 08028 Barcelona, Spain; 4 National Institute of Molecular Genetics (INGM), Via F. Sforza 35, 20122 Milan, Italy

**Keywords:** green route, silver nanoparticles, physico-chemical properties, inflammation response

## Abstract

The use of silver nanoparticles (Ag NPs) in the biomedical field deserves a mindful analysis of the possible inflammatory response which could limit their use in the clinic. Despite the anti-cancer properties of Ag NPs having been widely demonstrated, there are still few studies concerning their involvement in the activation of specific inflammatory pathways. The inflammatory outcome depends on the synthetic route used in the NPs production, in which toxic reagents are employed. In this work, we compared two types of Ag NPs, obtained by two different chemical routes: conventional synthesis using sodium citrate and a green protocol based on leaf extracts as a source of reduction and capping agents. A careful physicochemical characterization was carried out showing spherical and stable Ag NPs with an average size between 20 nm and 35 nm for conventional and green Ag NPs respectively. Then, we evaluated their ability to induce the activation of inflammation in Human Leukemic Monocytes (THP-1) differentiated into M0 macrophages using 1 µM and 2 µM NPs concentrations (corresponded to 0.1 µg/mL and 0.2 µg/mL respectively) and two-time points (24 h and 48 h). Our results showed a clear difference in Nuclear Factor κB (NF-κb) activation, Interleukins 6–8 (IL-6, IL-8) secretion, Tumor Necrosis Factor-α (TNF-α) and Cyclooxygenase-2 (COX-2) expression exerted by the two kinds of Ag NPs. Green Ag NPs were definitely tolerated by macrophages compared to conventional Ag NPs which induced the activation of all the factors mentioned above. Subsequently, the exposure of breast cancer cell line (MCF-7) to the green Ag NPs showed that they exhibited antitumor activity like the conventional ones, but surprisingly, using the MCF-10A line (not tumoral breast cells) the green Ag NPs did not cause a significant decrease in cell viability.

## 1. Introduction

Today, Ag NPs are the most used nanomaterials in several fields of application [1] due to their remarkable physicochemical properties [2,3]. Ag is known to have a sharp and strong plasmon resonance peak [4], showing high toxicity in cancer cells [5,6,7], extensively demonstrated in vitro [8] as well as antibacterial and antimycotic features [9,10]. However, the application of Ag NPs in clinical trials is often hindered by the activation of the inflammatory response [11]; in general, this occurs because the foreign material is recognized as an antigen [12]. Ideally, the NPs should not trigger the immune response in living organisms, when they are used as vectors or therapeutic agents [13]. The inflammation phenomenon in living organisms starts with the activation of different cells, such as neutrophils, basophils, eosinophils, monocytes and macrophages [14,15,16]. These cells release anti-inflammatory mediators through the action of inducible transcription factor NF-κB [17], which stimulates the expression of several pro-inflammatory cytokines genes, such as IL-6, IL-8 and TNF-α [18]. In addition, certain types of enzymes, such as COX are up-expressed as a result of the inflammation cascade [19,20,21]. A prolonged state of inflammation is responsible for neoplasm development [22] and other diseases [23], including rheumatoid arthritis [24], atherosclerosis [25], diabetes [26], and neurodegenerative pathologies [27]. Then, if the NPs are employed as drug carriers with specific surface functionalization able to bind cellular receptors, they would never reach the target due to the activation of the immune system [28]. In the case of biomedical applications, the immune system can be stimulated by the presence of synthetic residues and solvents used for the subsequent steps of NPs manufacture (washing, precipitation, etc.) that bind their surface [29,30]. In general, conventional synthetic methods used to obtain NPs require the use of toxic and hazardous agents [31,32,33]. To overcome this limitation, recent efforts have been made to replace such procedures with eco-friendly protocols [34,35,36], based on the use of plant or microorganism extracts as sources of reducing and capping agents [34,37]. The synthesis of metallic NPs mediated by plants can be obtained employing extracellular and intracellular routes [38]; moreover, phytochemicals previously isolated from plants are used to obtain NPs [39,40]. A large number of biomolecules, such as vitamins, phenols, proteins, flavonoids, saponins and aldehydes are involved in the reduction of Ag^+^ to Ag^0^ in aqueous solution [41,42] due to the presence of functional groups, such as –C–O–C–, –C–O–, –C=C–, and –C=O– [43]. The phytochemical profile of plants can vary according to the species; then, the size, shape and surface charge of NPs change together with pH, metal salt concentration, temperature and contact time [44]. The presence of biomolecules adsorbed on the NPs surface makes them safer for medical applications as therapeutic tools or antibacterial agents [45,46]. In the latter case, the capping agents were able to bind receptors of bacterial membranes damaging the respiratory system causing cell death [47,48]. Among different types of Mediterranean plants, *Laurus Nobilis* [49] is a valid source of polar molecules, such as polyphenols, which are demonstrated to have strong antioxidant and anti-inflammatory effects [50,51]. In addition, they take part in the biosynthesis of metallic NPs, such as Ag NPs acting as reducing and capping agents [52].

In this work, we synthesized Ag NPs using *Laurus Nobilis* extracts with an easy and reproducible one step route. First, a careful NPs characterization through different techniques, such as Transmission Electron Microscopy (TEM), RAMAN spectroscopy, UV-vis, Fourier-transform infrared spectroscopy (FTIR) and Dynamic Light Scattering (DLS) was carried out. Afterward, we evaluated the effects of Ag NPs in Human Leukemic Monocytes (THP-1) differentiated into macrophage-like cells (M0) mimicking native monocyte-derived macrophages. The obtained results were compared with those obtained exposing the cells to Ag NPs produced by the conventional synthetic method, based on sodium citrate and high temperature. The inflammation response was analyzed by measuring the amount of cytokines IL-6/IL-8 and the expression levels of TNF-α and COX-2. The nuclear translocation of NF-κB was evaluated by confocal microscopy and the morphometric parameters relating to nuclei and actin alterations using Fiji software. Our data showed that macrophages were less stimulated by exposure to green Ag NPs compared to those achieved by the conventional protocol. In addition, the potential antitumoral effects of green Ag NPs were explored on MCF-7 comparing the results with those obtained in non-tumoral cells (MCF-10A) showing high toxicity only in cancer cell lines.

## 2. Materials and Methods

### 2.1. Synthesis of Ag NPs

#### 2.1.1. Conventional Approach

Ag NPs were synthesized using an aqueous solution of tri-sodium citrate (1.4 mM) and 2.9 μM tannic acid. The mix was heated up to 60 °C in a silicon oil bath under reflux. After, the AgNO_3_ solution (0.6 mM) was added while stirring was heated up to the boiling point (ca. 120 °C) until the color turned to dark brown). Finally, the solution was washed with ethanol/water and centrifugated at 4000 rpm for 45 min to obtain NPs. Several washes were carried out to purify the NPs.

#### 2.1.2. Green Approach

##### Preparation of Leaves Extracts

*Laurus nobilis* leaves were washed with MilliQ to eliminate pollution and dried at room temperature for one day. Then, 10 g of leaves were sliced and transferred to a glass flask containing 100 mL of MilliQ water. The solution was boiled at 100 °C (20 min). After cooling, the solution was filtered by a cellulose membrane before use.

##### Synthetic Procedure

2.5 mL of leaves extract was added to 50 mL of AgNO_3_ (1 mM) and heated to 60 °C for about 45 min. During this time, the reaction color switched from light yellow to deep brown indicating the chemical reduction of Ag^+^ ions into Ag^0^ (pH 7). Finally, solutions were moved to centrifuge tubes and centrifuged at 4000 rpm for 1 h to achieve NPs.

### 2.2. Characterization of Conventional and Green Ag NPs

#### 2.2.1. Transmission Electron Microscopy (TEM) Analysis

Structural, morphological analyses and Selected Area Electron Diffraction (SAED) patterns were performed by Hitachi 7700 Transmission Electron Microscope (Hitachi High-Tech, Tokyo, Japan), operating at 100 kV; 10 µL of the two different Ag NP solutions were dropped onto standard 400-mesh carbon-supported copper grids and air-dried overnight. Statistical analysis of Ag NPs size was obtained by Gatan Digital Micrograph software (Las Positas Blvd., Pleasanton, CA, USA).

#### 2.2.2. Dynamic Light Scattering (DLS) and ζ-Potential Analyses

The DLS and ζ-potential measurements were recorded by a Zetasizer Nano-ZS, equipped with a HeNe laser (4.0 mW) working at 633 nm detector (ZEN3600, Malvern Instruments Ltd., Malvern, UK) in aqueous solutions (25 °C, pH 7).

#### 2.2.3. UV-Vis Analysis

In order to acquire the absorption spectra (in the spectral range 300–800 nm) of the two different Ag NPs (conventional and green), a Varian Cary 5 spectrophotometer (ZEN3600, Malvern Instruments Ltd., Malvern, UK) equipped with a quartz cuvette of 10 mm path length was used at room temperature.

#### 2.2.4. Raman Scattering Analysis

Raman scattering measurements were obtained in back-scattering geometry with a RENISHAW spectrometer (Wotton-under-Edge, Gloucestershire, UK) coupled to a LEICA metallographic microscope. Excitation radiation was given out by an argon ion laser operating at a wavelength of 514.5 nm and an incident power of 10 mW to avoid the thermal effects provided by the excitation. Raman shifts were calibrated using silicon (111) reference spectra after each measurement.

#### 2.2.5. Fourier Transform Infrared (FTIR) Spectroscopy

Jasco-670 Plus FTIR spectrometer (Jasco, Tokyo, Japan) was used to measure the FTIR spectra over a range of 800–4000 cm^−1^ of the two types of Ag NPs at a resolution of 4 cm^−1^.

### 2.3. THP-1 Culture and Differentiation

The Human Leukemic Monocytes (THP-1) (ATCC-TIB-202) were grown in Roswell Park Memorial Institute medium (RPMI-1640) supplemented with 2 mM l-glutamine, 25 mM HEPES (Sigma-Aldrich, Dorset, UK), 10% (*v*/*v*) fetal bovine serum (FBS, Sigma-Aldrich, Dorset, UK), 1% (*v*/*v*) penicillin-streptomycin (Sigma-Aldrich, Dorset, UK), and 0.1% (*v*/*v*) amphotericin B (Sigma-Aldrich, Dorset, UK). Before starting the experiments, THP-1 were differentiated into a mature macrophage-like state (M0-macrophages) by incubation with 10 ng/mL of phorbol 12-myristate 13-acetate (PMA, Sigma-Aldrich, Dorset, UK) for 48 h in a humidified atmosphere and standard conditions (95 % air and 5% CO_2_, at 37 °C).

### 2.4. MCF-7 and MCF-10 Cell Culture

Human breast cancer cell (MCF-7) (ATCC-HTB-22) and non-tumorigenic epithelial breast cell line (MCF-10) (ATCC-CRL-10317) were cultured in high glucose Dulbecco’s Modified Eagle Medium (DMEM) with 50 μM of glutamine, 10% FBS, 100 U/mL of penicillin and 100 mg/mL of streptomycin. Cells were maintained in a humidified controlled atmosphere with a 95% to 5% ratio of air/CO_2_, at 37 °C.

### 2.5. Viability Assay of THP-1, MCF-7 and MCF-10

The THP-1, MCF-7 and MCF-10 cells were seeded at a concentration of 5 × 10^3^ cells per well in 96-well plates. THP-1 was differentiated as described above. After 24 h of stabilization, Ag NPs stock solutions (conventional Ag NPs and green Ag NPs) were added to the culture medium at 1 µM (0.1 µg/mL) and 2 µM (0.2 µg/mL) for 24 h and 48 h. After these times, cell viability was calculated using a standard WST-8 assay (Sigma-Aldrich, Dorset, UK) following the procedure described in [53]. Data were expressed as mean ± SD. To calculate the half maximal inhibitory concentration (IC50), i.e., concentration causing a 50% inhibition compared to the controls, data were fitted to a regression model equation for a sigmoid curve: y = max/[1 + e − (x − IC50)/b)] + min, where: max represented the maximal response measured, b represented the slope of the curve and min the minimal response.

### 2.6. NPs Concentration and Uptake Determination by Elemental Analysis

The concentrations of the conventional Ag NPs and green Ag NPs were calculated by elemental analysis using an ICP-OES Perkin Elmer AVIO 500 (Waltham, MA, USA) following the procedure described in [3].

In order to measure the cellular content of NPs internalized by cells, 1 × 10^5^ of THP-1 (after differentiation), cells were seeded in 1 mL of the medium in a 6-well plate. After 24 h, the cell culture medium was discarded and restored with 1 μM and 2 μM of Ag NPs (conventional and green) dissolved in fresh medium for 24 h and 48 h. At the end of time points, NPs were removed, and cells were washed several times with Phosphate Buffered Saline (PBS, Sigma-Aldrich, Dorset, UK). After detachment, cells were counted by an automatic cell counting chamber to obtain 360,000 cells dispersed in 200 μL of MilliQ. The digestion process was made by Nitric Acid (>90%, Sigma-Aldrich, Dorset, UK) for one week. The solutions were analyzed to evaluate the Ag amount after dilution with MilliQ water, using an ICP-OES Perkin Elmer AVIO 500 (Waltham, MA, USA).

### 2.7. Interleukins 6, 8 (IL-6, IL-8) Quantification by ELISA Assay

Cytokines IL-6 and IL-8 were measured by an enzyme-linked immunosorbent assay (ELISA) on differentiated THP-1 exposed to 1 μM and 2 μM of conventional and green Ag NPs for 24 h and 48 h. After a centrifugation step at 2000× *g* for 10 min, the supernatants from the cultures containing 0.5 × 10^6^ cells/mL in a final volume of 1 mL were collected and stocked at −80 °C until the analyses. Human IL-6 and IL-8 ELISA kits (Abcam, Cambridge, UK) were employed, following the manufacturing protocol and the quantifications were spectrophotometry carried out.

### 2.8. TNF-α and COX-2 Expression Levels by Western Blot Analysis

Western blots were performed on THP-1 cell extracts following the treatment by conventional and green Ag NPs (1 μM and 2 μM) for 48 h. The protein quantity of the cellular fraction was measured by a Bradford protein assay using BSA protein as a standard [54]. The separation of proteins was carried out by 12.5% Sodium Dodecyl Sulfate polyacrylamide gel electrophoresis (SDS-PAGE 12.5%). The blot was then blocked using 5% of non-fat dried milk in Tris-buffered saline with 0.05% Tween 20 (TBST) at room temperature for 1 h. After this time, the incubation with primary antibody (rabbit anti-tumor necrosis factor (TNF)-α polyclonal antibody (17H1L4, Thermo fisher Scientific, Waltham, MA, USA) and mouse antihuman COX-2 (Cayman Chemicals, Ann Arbor, MI, USA) was carried out on a shaker at 4 °C overnight. After this experimental step, the membrane was washed and incubated with a horseradish peroxidase-conjugated secondary antibody for 2 h at room temperature. Densitometry was used to measure the relative band intensities, that were normalized on the untreated samples to Glyceraldehyde-3-phosphate dehydrogenase (GAPDH) signal.

### 2.9. NF-κB Signaling Imaging, Quantification Assay, and Morphometric Parameters

NF-κB signaling imaging was performed using a Confocal Laser Scanning Microscope (CLSM, Leica SP8, Milton Keynes, UK). After THP-1 differentiation, the M0 macrophages were exposed to 1 µM and 2 µM of conventional and green Ag NPs for 24 h and 48 h in a humidified atmosphere, with 95% air and 5% CO_2_, at 37 °C. At the endpoint, cells were washed with PBS (Sigma-Aldrich, Dorset, UK) and fixed using 3.7% formaldehyde (Sigma-Aldrich, Dorset, UK) for 10 min at room temperature. Then, TritonX (0.2%) (Sigma-Aldrich, Dorset, UK) was used to permeabilize the cells prior to performing the further experimental immunostaining procedure using NF-κB p65 Antibody (F-6) and FITC (Santa Cruz Biotechnology Inc., Heidelberg, Germany) diluted in 1% BSA (overnight, 4 °C). Then, the fixed samples were washed with PBS and labeled with DAPI (Sigma-Aldrich, Dorset, UK) for nuclei visualization.

The NF-κB nuclear translocation imaging study was carried out by co-localization (Pierce’s coefficient values) of the NF-κB and nucleus fluorescence intensity signals using the Fiji ImageJ software (version 2.0, National Institutes of Health, MD, USA).). The cell membrane was marked using a CellMask™ Deep Red Plasma Membrane Stain (Thermo Fisher Scientific, Waltham, MA, USA).

The morphometric quantifications (actin and nuclear density) were measured on confocal acquisitions using ImageJ 1.47 software by means of Integral Density tools [55]. The value of morphometric parameters was expressed as the mean value and its relative standard deviation calculated on 30 different cells for each treatment.

### 2.10. Statistical Analysis

Statistical analyses were performed using OriginPro (version 8.1). The differences between two groups were calculated by a two-tailed Student’s-test. The comparison between three and more groups was analyzed by one-way or two-way ANOVA multiple comparisons, respectively. The differences were statistically significant when * *p* < 0.05, ** *p* < 0.01.

## 3. Results and Discussion

**Figure 1:**
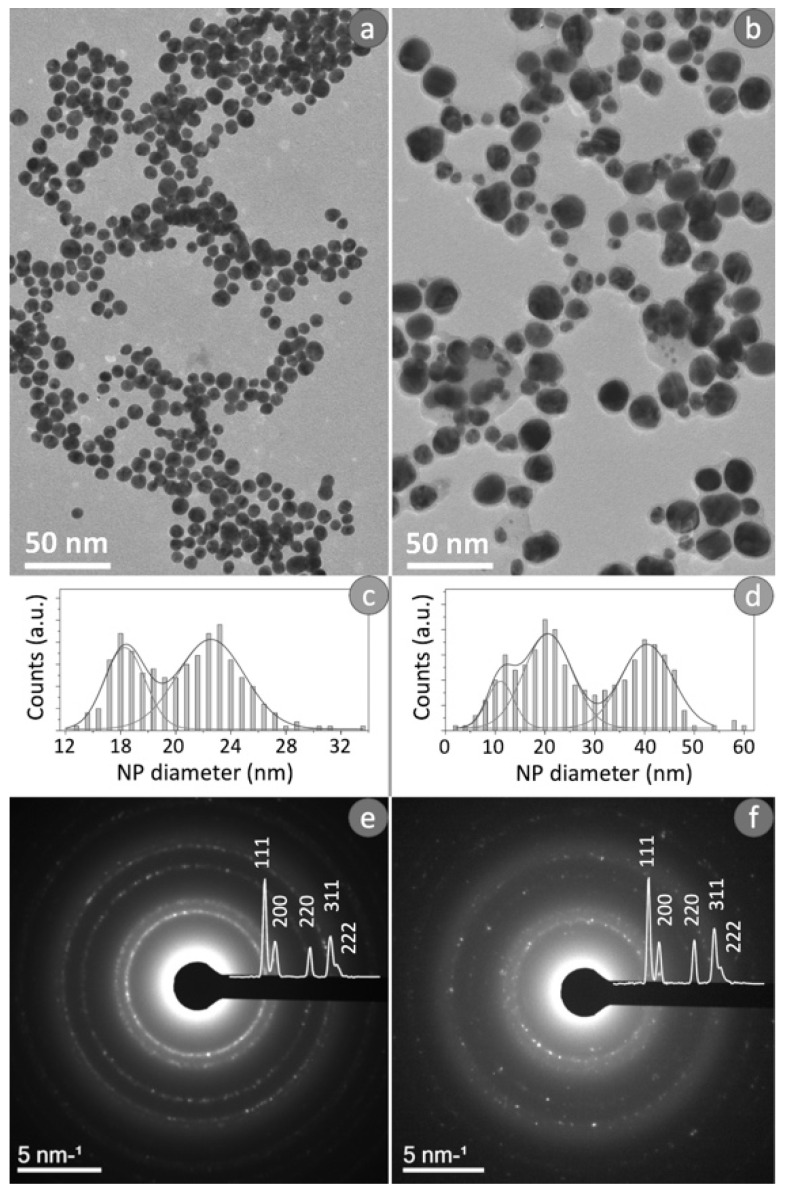
Typical bright field TEM images obtained from conventional Ag NPs (**a**), related size distribution (**c**) and SAED pattern (**e**). Typical bright field TEM image recorded from green Ag NPs (**b**), related size distribution (**d**) and SAED pattern (**f**).

Morphological and structural characterization of Ag NPs obtained from conventional and green Ag NPs were reported in Figure 1. In detail, Figure 1a,b showed typical bright-field TEM images of two kinds of Ag NPs. The observation of these images revealed the difference in the size of the NPs obtained from the two synthetic processes. The conventional route permitted to achieve spherical Ag NPs with a slight variation in size (12–30 nm in diameter); whereas the NPs obtained by green synthesis, were larger showing an average diameter ranging from 6 to 50 nm.

To study the size distribution for the conventional Ag NPs (Figure 1c) and the green counterpart (Figure 1d), TEM images were analyzed using Gatan’s Digital Micrograph software. The distribution obtained for the conventional Ag NPs (Figure 1c) presented a clear bimodal distribution, then the data were fitted by two Gaussians: the two maximum values were obtained in correspondence to (16 ± 2) nm and (22 ± 4) nm. Meanwhile, the size distribution of the green Ag NPs reported in Figure 1d exhibited a clear trimodal distribution; therefore, these data were fitted by three Gaussians in order to calculate the three peak values, which resulted equal to (10 ± 4) nm, (20 ± 8) nm and (40 ± 10) nm.

Figure 1e,f showed the typical SAED patterns recorded by the two types of NPs obtained. Distinctive features of the SAED patterns were diffraction maxima arranged along concentric rings determined by the random arrangement of the nanocrystals. The diffraction patterns displayed the superimposed intensity profile. The diffraction peaks due to the lattice planes (111), (200), (220), (311) and (222) clearly indicated the presence of a face-centered cubic phase (fcc) of metallic silver (JCPDS File No. 04-0783 from ASTM 1999).

The size of NPs was also measured using DLS whereas the surface charge was assessed by ζ-potential (Table 1). In water, the two colloidal solutions were stable, and sizes were (20 ± 3) nm and (32 ± 6) nm for conventional Ag NPs and green Ag NPs respectively. The ζ-potential was negative for both types of NPs, i.e., (−30 ± 3) mV and (−35 ± 4) mV. In the DMEM and RPMI, the presence of proteins and other nutrients made the size of NPs larger and more negative. In general, a high value of ζ-potential confers greater stability to colloidal systems, as electrostatic repulsions are generated which prevent the aggregation of dispersed particles.

**Figure 2:**
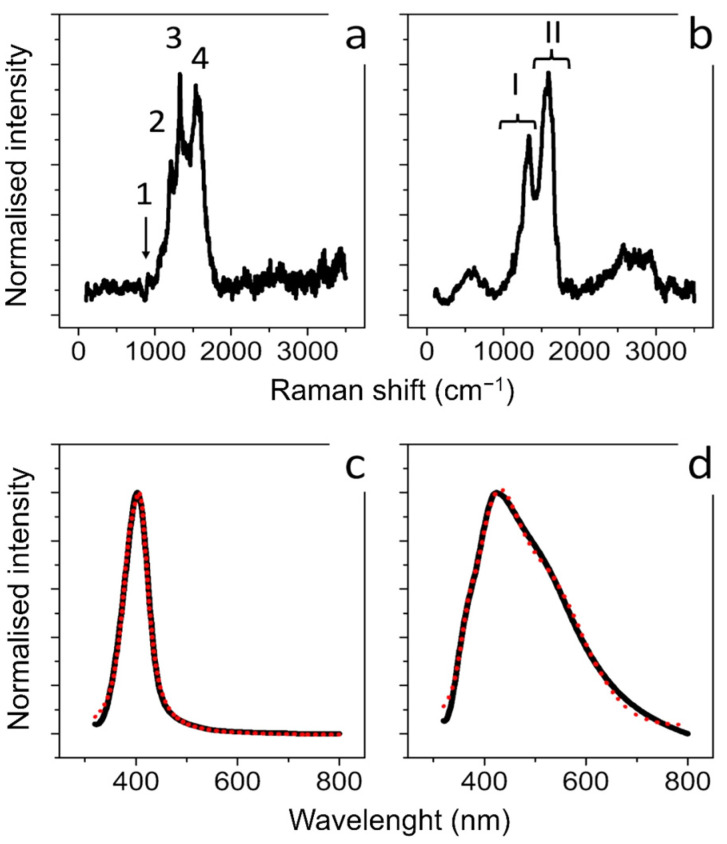
Raman (**a**) and UV-V is absorbance spectra (**c**) obtained from conventional Ag NPs synthesized using citrate: 1, 2, 3, and 4 in (**a**) marked the peaks related to n(C–COO), n s(COO), n s(COO) and n as(COO) of citrate; Raman (**b**) and UV-VIS absorbance spectra (**d**) obtained from green Ag NPs. (I), (II) in (**b**) marked the peaks related to C=C and C=O bonds. In (**c**,**d**), the black lines represented the experimental optical absorption and the dashed red curve the experimental fit.

Raman spectroscopy was applied to obtain information regarding the molecular properties of Ag NPs obtained by the two synthetics routes. Figure 2a,b showed the Raman spectra carried out on the aqueous solutions of conventional and green Ag NPs, respectively. Regarding Ag NPs synthesized with citrate the peaks at 930 cm^−1^, 1300 cm^−1^, 1370 cm^−1^ and 1575 cm^−1^ (marked 1, 2, 3, and 4, respectively, in Figure 2a) corresponded to the typical Raman spectrum of citrate adsorbed onto the surface of conventional Ag NPs. The bands are related to n(C–COO), n s(COO), n s(COO) and n as(COO) of citrate, respectively [56,57]. The green Ag NPs showed different results: the main feature of the Raman spectrum obtained (Figure 2b) showed two broad bands at about 1350 cm^−1^ and 1565 cm^−1^ (marked I and II respectively) originating from the superposition of a series of vibrational modes attributable to stretching vibrations of C=C and C=O bonds [58]. This supported the presence of polyphenols involved as reducing and capping agents in Ag NPs formation [59]. In Figure 2c,d we reported the typical absorbance spectra acquired on conventional Ag NPs (Figure 2c) and green Ag NPs (Figure 2d). A well-defined peak and a broad peak at about 410 nm were obtained from both types of Ag NPs. These peaks were clearly due to localized plasmon resonance of Ag NPs dispersed in water. The absorption spectra were analyzed according to the light scattering theory described by Mie and the free electron Drude’s theory [60] to explain the characteristic absorption peak due to localized plasmon resonance. In detail, the absorption coefficient (*α_j_*) due to particles dispersed in the dielectric medium [61], is given by:

(1) $$\alpha_{j}=\frac{18\pi f_{j}\varepsilon_{m}^{\frac{3}{2}}\varepsilon_{2j}}{\lambda {\left(\varepsilon_{1j}+2\varepsilon_{m}\right)}^{2}+\varepsilon_{2j}^{2}}$$

where *f_j_* is the volume fraction of the metal particles (i.e., Ag NPs) with diameter *d_j_*, *λ* is the photon wavelength, *ε_m_* is the dielectric constant of medium (i.e., water), and *ε_j_* = *ε*_1*j*_ + *iε*_2*j*_ is the dielectric complex function of the Ag NPs.

The Mie scattering theory demands the sphericity of materials, which need to have a constant diameter. To consider the polydispersion of our Ag NPs, it is convenient to define the total absorption coefficient (*α*):

(2) $$\alpha =\underset{j}{\sum }\alpha_{j}$$

Considering the volume fraction of the Ag NPs with *d_j_* diameter:

(3) $$fw_{j}=f_{j}$$

where *w_j_* is the weight factor for particles with diameter *d_j_* and is given by:

(4) $$w_{j}=\frac{d_{j}^{3}n_{j}}{\sum_{j}d_{j}^{3}n_{j}}$$

*n_j_* being the number of NPs with diameter *d_j_*. Hence, it can be easily to obtain the size-dependent dielectric function as:

(5) $$\varepsilon_{j}\left(\omega \right)=\varepsilon_{bulk}\left(\omega \right)-\frac{{\left(\frac{\omega_{P}}{\omega }\right)}^{2}}{1+{\left(\frac{2v_{F}}{\omega d_{j}}\right)}^{2}}+i\frac{2v_{F}\omega_{P}^{2}}{\omega^{3}d_{j}}$$

Here, *ω*, *v_F_* and *ε_bulk_* indicate the photon frequency, the Fermi velocity and the dielectric constant relative to interbond transitions, respectively.

To confirm the size distribution data obtained by TEM image analysis, the experimental absorption data were fitted, imposing *d_j_* and *f_j_* as fit parameters. The constant values relative to plasma frequency and Fermi velocity were fixed at *ω_P_*_0_ = 3.7 × 10^15^ rad/s and *v_F_* = 1.38 × 10^8^ cm/s, respectively [62].

The theoretical fittings, reported in Figure 1c (full line) were obtained both for a volume fraction of 30% of NPs having a mean size of (10 ± 4) nm, and a volume fraction of 70% of NPs with a mean size of (25 ± 8) nm. Similarly, as regards Figure 1d, the theoretical fitting was obtained for a volume fraction of 20% of NPs having a mean diameter of (10 ± 2) nm, a volume fraction of 20% of NPs having a mean size of (20 ± 4) nm and a volume fraction of 60% of NPs with (50 ± 10) nm. The NPs size dispersion derived from the absorption fit data was in good agreement with the NPs size distribution obtained from analysis performed on TEM acquisitions.

**Figure 3:**
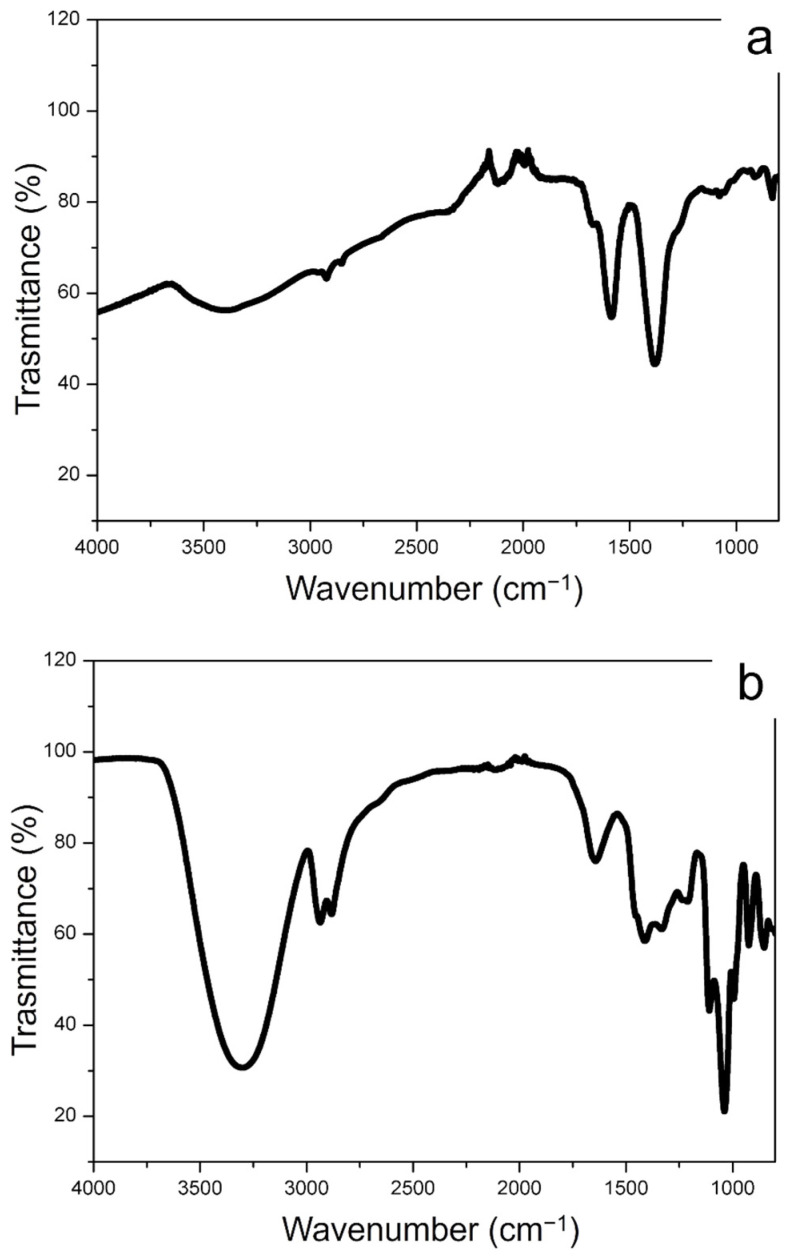
FTIR spectra obtained from conventional Ag NPs (**a**) and green Ag NPs (**b**).

The FTIR spectra of conventional Ag NPs and green Ag NPs were observed in Figure 3a,b that complete the characterization obtained by RAMAN spectroscopy. In Figure 3a, the presence of citrate on the conventional Ag NPs surface was proven by the peaks corresponding to R–CO_2_ and C–O stretching in the range between 1560 cm^−1^ and 1300 cm^−1^. The peaks of 3200 cm^−1^ were ascribed to the stretching vibrations of O–H. The FTIR spectra of the Ag NPs synthetized from Laurus Nobilis, showed the broad band peak at ca. 3200 cm^−1^ and the peaks at ca. 2927 cm^−1^, 2854 cm^−1^, 1651 cm^−1^ and 1023 cm^−1^ corresponded to O-H and C-H vibration of alcohols and polyphenols. We observed other representative peaks in the low wavenumber region in the range of 1600–1400 cm^−1^ associated with COO- stretching of aldehydes and ketones surrounding the Ag NPs surface. These vibrations clearly showed the presence of polyphenols compounds as capping agents of green Ag NPs.

**Figure 4:**
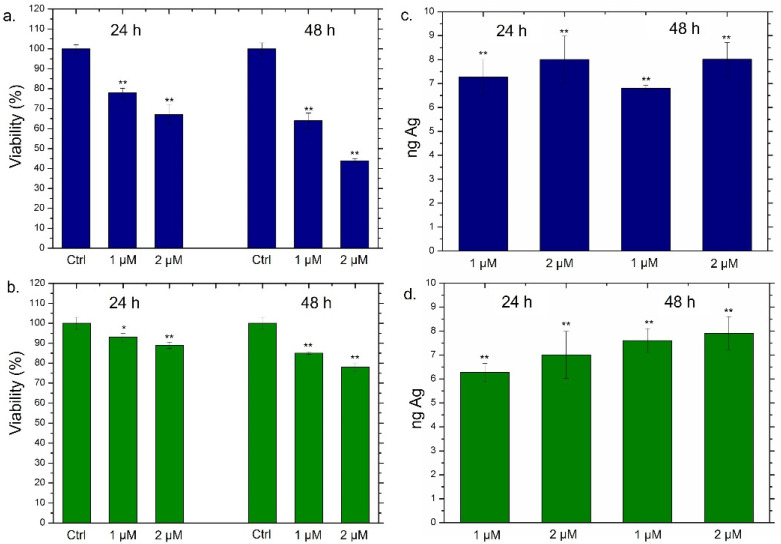
(**a**,**b**) Viability assay performed on THP-1 cell lines exposed to 1 µM and 2 µM of conventional Ag NPs (**a**) and green Ag NPs (**b**) after 24 h and 48 h. The viability of cells exposed to NPs was normalized to control cells (untreated). As a positive control (P), 5% of DMSO was used (data not shown). Data reported as the mean ± SD from three independent experiments are considered statistically significant, compared with the control (n = 8) for *p*-value < 0.01 (<0.01 **) and < 0.05 (<0.05 *). (**c**,**d**) Uptake of conventional Ag NPs (**c**) and green Ag NPs (**d**) in THP-1 cell lines at concentrations of 1 µM and 2 µM for 24 h and 48 h. Untreated cells represented the controls (values = 0, data not shown). The data were reported as the mean ± SD from three independent experiments. Data were statistically significant in comparison to exposed cells vs. control cells (ag content is equal to 0) for *p*-value < 0.01 (<0.01 **).

Following the accurate characterization of Ag NPs obtained through the two synthetic approaches, we went to evaluate the viability of THP-1 cells incubated with two different concentrations of Ag NPs (1 µM and 2 µM) at 24 h and 48 h (Figure 4).

For this purpose, THP-1 was differentiated into the primary non-activated macrophage (M0) phenotype by PMA incubation. As expected, the conventional Ag NPs induced a significant viability reduction; such effect was strictly dependent both on time and on the dose. In particular, the highest dose (2 µM) triggered the vitality decrease of about 60% after 24 h and 44% after 48 h of Ag NPs exposure (Figure 4a). The results concerning the impact of green Ag NPs on macrophages were completely different; in this case, we observed slight cell death (the viability was reduced by about 20%) only after 48 h using 2 µM of concentration (Figure 4b). To understand whether the different toxic response was correlated to different macrophages’ uptake, the cellular internalization of conventional Ag NPs and green Ag NPs was assessed by ICP-OES. The amount of internalized Ag NPs was evaluated in terms of Ag concentrations measured after exposure to 1 µM and 2 µM of conventional and green NPs at two time points. As reported in Figure 4c,d, no substantial differences in the internalization process were noted using the two different types of NPs. As a matter of fact, when cells were exposed to the highest concentration for 48 h, we observed an Ag amount of about 8 ng both in the case of conventional Ag NPs (Figure 4c) and green Ag NPs (Figure 4d). Therefore, we concluded that the different toxicity was not due to a different uptake rate. Sure enough, the two types of NPs appeared to be similar in diameter when dispersed in the cell culture medium RPMI-1640. Therefore, the endocytosis mechanism was comparable.

**Figure 5:**
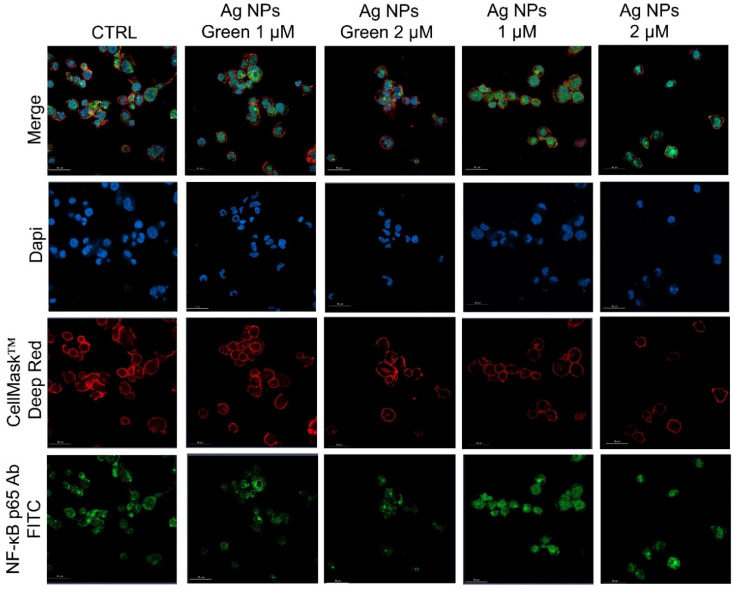
Representative confocal images of untreated macrophages (M0, control) and macrophages exposed to the 1 µM and 2 µM of green Ag NPs and conventional Ag NPs for 24 h. The cells were fixed and then stained. The nuclei were labeled with DAPI (blue), Actin cytoskeleton with CellMask™ (red), and NF-κB with NF-κB p65 Antibody (F-6) FITC (green intensity signal). Scale bar is 50 µm.

**Figure 6:**
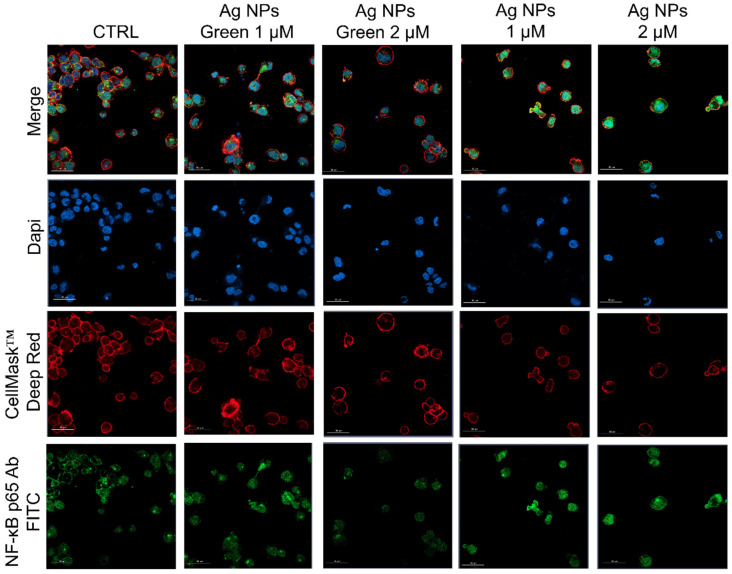
Representative confocal images of untreated macrophages (M0, control) and M0 exposed to the 1 µM and 2 µM of green Ag NPs and conventional Ag NPs for 48 h. The cells were fixed and then labeled as described in Figure 5. Scale bar is 50 µm.

Subsequently, the regulation of inflammatory response was assessed by observing the NF-κB nuclear translocation, which plays a key role in the activation and differentiation of inflammatory cells [63,64]. NF-κB family represents a class of inducible transcription factors [65], which are physiologically localized in the cytoplasm by inhibitory proteins. When an inflammation phenomenon is induced, NF-κB is activated by translocation to the nucleus where it binds specific DNA sequences involved in the activation of cytokines and other pro-inflammatory elements [66]. In Figure 5 and Figure 6 were reported the confocal acquisition of THP-1 cells exposed to 1 µM and 2 µM of conventional and green Ag NPs for 24 h and 48 h. The actin network, nuclei and NF-κB were labeled with specific markers. In control cells, we observed the green fluorescence related to NF-κB in the cytoplasm already after 24 h (Figure 5) and 48 h (Figure 6). In the merged images it was possible to clearly see the different compartments. The treatment with green Ag NPs caused only moderate nuclear translocation of NF-κB, compared to the untreated cells, indicating that the cells did not undergo severe inflammation processes. This result was similar using the two concentrations of green Ag NPs. In contrast, when the macrophages were exposed to conventional Ag NPs, NF-κB translocated into the nuclei causing a merged fluorescence between DAPI (blue) and NF-κB p65 Ab-FITC in a dose-dependent manner.

**Figure 7:**
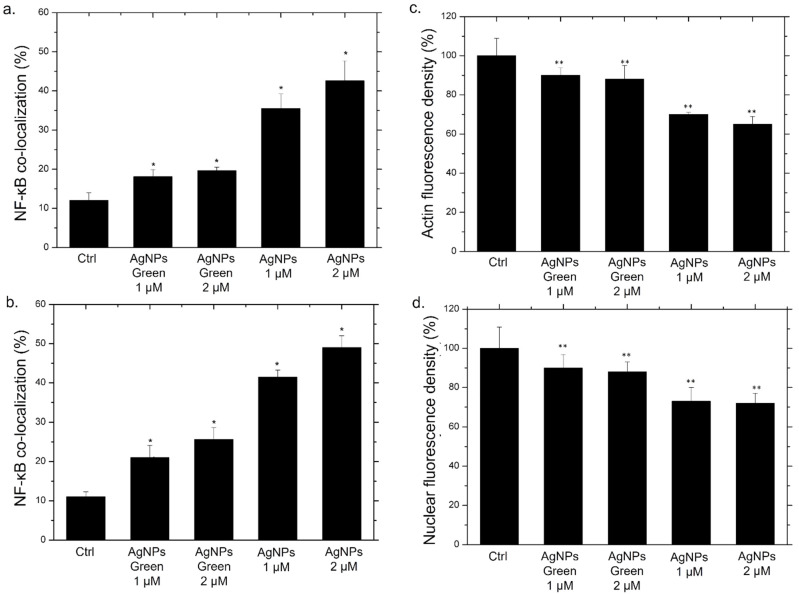
Co-localization analysis of the merged fluorescence signals on confocal images due to the NF-κB translocation from the cytoplasm to the nucleus (merged blue/green fluorescence intensity signal) after 24 h (**a**) and 48 h (**b**). The data are expressed as the mean SD (5 images for n = 2) and they were considered statistically significant for a * *p* < 0.01 (<0.01 **) and *p* < 0.05 (<0.05 *). Mean values and their respective standard deviation of actin density fluorescence (**c**) and nuclear density fluorescence (**d**) calculated on confocal acquisitions of THP-1-NPs treated for 48 h.

In addition to qualitative analysis, we also carried out the quantification of co-localization within the nucleus by the Pearson coefficient on confocal images (Figure 7a,b).

After 24 h of incubation with the two types of NPs, green Ag NPs did not induce an evident translocation, showing an average percentage of around 25% for both concentrations. On the other hand, the co-localization data relative to conventional NPs exposure, revealed a drastic increase in percentage: with the co-localization rate becoming equal to 43% using the highest concentration, which was almost double the value recorded for green NPs (Figure 7a). After 48 h, the translocation of NF-κB becomes more evident, reaching values of about 50%, whereas in cells exposed to green NPs, no considerable changes were observed (Figure 7b).

It was demonstrated that the activation of NF-κB induces a series of cascade events. Among the countless biochemical pathways, the alteration of actin [67,68] and the nuclear chromatin amount were demonstrated [69,70]. Then, analysis on actin and nuclei fluorescence density was carried out using ImageJ software. In particular, the integrated fluorescence density, evaluated on blue and red channels of fluorescence images, was a direct indicator of the local concentration of cytoskeletal actin and nuclear chromatin amount, respectively. In Figure 7c,d we reported the values obtained for this parameter as a percentage with respect to the control, indicated as 100%. The actin fluorescence integrated density measured on macrophages incubated for 48 h with the conventional Ag NPs underwent a drastic reduction, reaching average values of 63% (Figure 7c). Contrary, the exposure to green NPs did not induce evident effects; the fluorescence integrated density value was reduced by only 10% compared to the control. A similar outcome was evident regarding the nuclear density. In this case, the reduction of fluorescence was particularly evident using conventional NPs (Figure 7d). In close agreement with results obtained by previous experiments, the internalization of conventional NPs by cells induced actin and nuclei damage underlining the greater predisposition of these kinds of NPs to induce an inflammatory response compared with their green counterpart.

**Figure 8:**
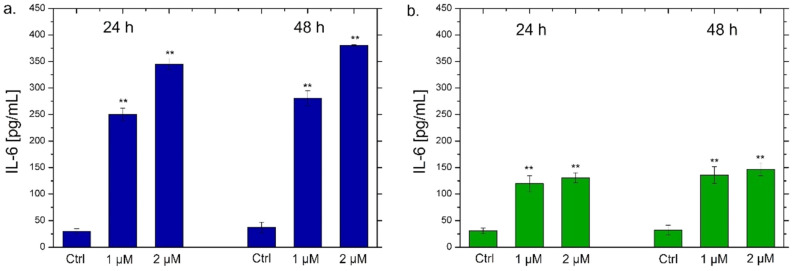

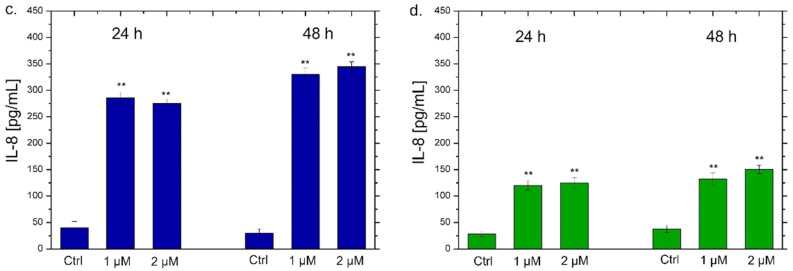
IL-6 (**a**,**b**) and IL-8 (**c**,**d**) levels expressed as pg/mL measured after exposure of THP-1 cells to conventional Ag NPs and green Ag NPs (1 µM and 2 µM) for 24 h and 48 h. The cytokine amount was detected in supernatants derived from the control and the treated cells by ELISA assay. The data were reported as the mean ± standard deviation of three separate experiments. *p*-value < 0.01 (<0.01 **) compared to the control of each time point.

NF-κB triggers the secretion of different types of cytokines, in particular IL-6 and IL-8, which are small proteins that regulate the inflammation pathways [71]. Each cytokine has a specific role. IL-6 acts as a multifunctional cytokine, both pro-inflammatory and anti-inflammatory [72]; it is secreted by T-lymphocytes and macrophages to stimulate the immune response, for example during an infection or following trauma and tissue damage [73]. Therefore, the persistent and dysregulated production of IL-6 plays a pathological role in various autoimmune and chronic inflammatory diseases [74]. IL-8 is a chemokine produced by macrophages and other types of cells, such as epithelial cells [75]. It is a chemotactic factor for neutrophils inducing chemotaxis of target cells (neutrophils and granulocytes), which migrate to the infection site [76]. In addition, it is a strong angiogenesis promoter [77]. In the light of this, we verified the possible secretion of these two classes of cytokines by THP-1 cells by ELISA assay to confirm the data obtained by confocal analysis. As observed in Figure 8, a different trend was noticed using the two types of Ag NPs. In particular, the exposure to the conventional Ag NPs for 24 h and 48 h strongly increased the secretion of IL-6 and IL-8 (Figure 8a,c). In detail, values of about 270 pg/mL and 380 pg/mL of IL-6 were secreted after incubation with 1 µM and 2 µM of conventional Ag NPs respectively after 48 h (Figure 8a). At the same time point, similar data were achieved measuring the amount of IL-8 at the same concentration of NPs: 330 pg/mL and 350 pg/mL. Considering that the untreated cells showed values of about 25 pg/mL, it was quite intuitive to conclude that this type of NPs promoted a strong activation of cytokines.

Similar results were obtained in recent works where conventional Ag NPs were used on THP-1 cells. In particular, polyvinylpyrrolidone (PVP)-coated Ag NPs with a size less than 100 nm triggered the up-regulation of pro-inflammatory cytokines gene expression in macrophages and also in primary blood monocytes [78]. In murine macrophage cell lines (RAW) biomarkers associated with inflammation were stimulated using ca. 25 μg/mL of Ag NPs coated with PVP [79]. In addition, our results showed that the toxic behavior was evident at low doses of conventional Ag NPs. This evidence was demonstrated in our previous work in which the same plant extract was used to achieve Au NPs tested on THP-1 cells [80]. In addition, a lot of studies reported the employment of similar low doses of Ag NPs in vitro [81] and in vivo [82,83,84]. showing high toxicity and inflammation activation in different cell lines.

Using green Ag NPs, the data were different because the stimulation of IL-6 and IL-8 secretion was very low. The maximum value was about 150 pg/mL both for IL-6 and IL-8 after the incubation with green Ag NPs (2 µM) for 48 h. These results confirmed the low toxic effects of green Ag NPs on macrophages. Although a minimal activation of cytokines production was recorded, it was not comparable with NPs obtained with the conventional approach. These results were in line with those obtained by a recent work in which Ag NPs obtained by *Salvia coccinea* leaf extracts were anti-inflammatory agents, that efficiently inhibited inflammation in THP-1 cells [85].

**Figure 9:**
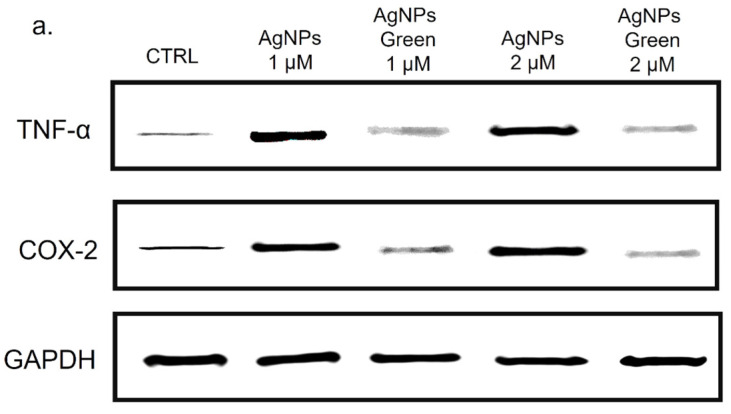

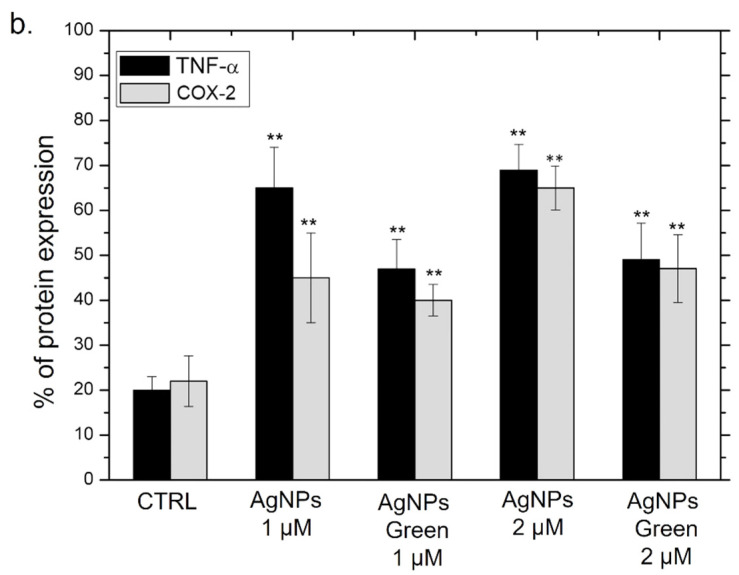
Western blot (**a**) and densitometric analysis (**b**) of TNF-α and COX-2 expression on THP-1, after 48 h of exposure to 2 µM of conventional and green Ag NPs. The reported data are estimated as an average of five independent experiments ± SD and they are considered statistically significant with *p*-value < 0.01 (<0.01 **).

Besides the IL-6 and IL-8, we analyzed the Cyclooxygenase-2 (COX-2) production due to its critical role in the pathogenesis of several inflammatory diseases [86] and cancer [87]. COX-1, COX-2 and COX-3 are three iso-enzymatic forms of prostaglandin-endoperoxide synthase [88]; COX-2 is usually expressed at low levels in several tissues and cells; therefore, it can be strongly induced by some kinds of cytokines, such as TNF-α [89]. On the other hand, TNF-α is involved in systemic inflammation stimulating the acute phase reaction [90]. It is mainly produced by macrophages, CD4^+^ T lymphocytes, NK cells, neutrophils, mast cells, eosinophils, and neurons showing a critical contribution to rheumatoid arthritis pathogenesis and other diseases [91]. Then, the expression of COX-2 and TNF-α levels in THP-1 cells were studied using immunoblot analysis (Figure 9a) followed by the incubation with conventional and green Ag NPs for 48 h at 1 and 2 µM of concentration. The densitometric analysis clearly showed an up-expression of both COX-2 and TNF-α in THP-1 cells exposed to conventional Ag NPs, in close agreement with the results obtained in the previous experiments. Using 2 µM of Ag NPs the percentage of expression of TNF-α reached 70% using conventional NPs versus 50% of green NPs compared to the control cells (values of about 20%). The same trend was clearly observed for COX-2. Additionally, in this case, the percentage of expression upon conventional Ag NPs was about 65% whereas the green counterpart value was 48% with respect to the untread cells (ca. 22%) (Figure 9b).

**Figure 10:**
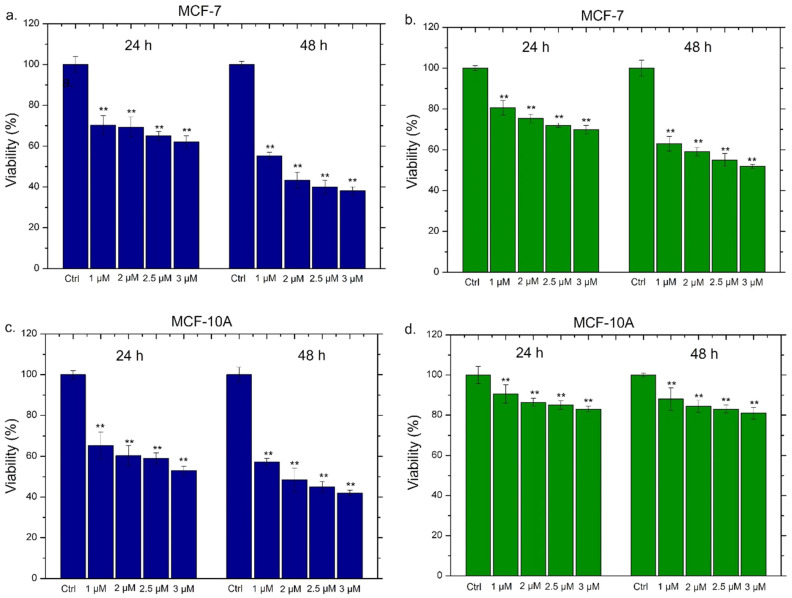
Viability assay performed on MCF-7 and MCF-10A cell lines exposed to 1 µM, 2 µM, 2.5 µM and 3 µM of conventional Ag NPs (**a**,**c**) and green Ag NPs (**b**,**d**) after 24 h and 48 h. The viability of cells exposed to NPs was normalized to untreated cells (control). The positive control was represented by cells incubated with 5% DMSO (data not shown). Data reported were the mean ± SD from three independent experiments compared with the control (*n* = 8) for *p*-value < 0.01 (<0.01 **).

All the experiments were consistent with each other, leading to the conclusion that conventional Ag NPs were more able to induce an inflammatory response in macrophages. The green Ag NPs, on the contrary, induced an inflammation showdown maintaining the macrophages’ health: this is probably due to the presence of polyphenol capping. These results were promising starting points to apply green Ag NPs in vivo as anticancer agents without activating the immune system response. Then, their anticancer outcomes on breast cancer cell lines (MCF-7) were evaluated analyzing the cell death. Since toxicity on healthy cells could be a problem in view of possible in vivo treatments, we assessed the viability also on the breast cells (MCF-10A), which were the non-tumorigenic counterpart. The data were compared with those obtained with the conventional Ag NPs; in the histograms reported in Figure 10, the general trend was immediately evident. We also tested the effect of Ag NPs at higher concentrations (2.5 µM and 3 µM) in order to measure the IC50 values (Table 2).

The conventional Ag NPs induced toxicity both in MCF-7 (Figure 10a) and MCF-10A (Figure 10c). In detail, in MCF-10A, the exposure to 2 µM of conventional NPs for 48 h triggered a reduction in cell viability of about 50%. The increase of the doses (2.5 µM and 3 µM) showed that this effect was dose and time dependent. Meanwhile, the green Ag NPs exhibited different trends in MCF-7 and MCF-10A. In the tumoral cell lines, they promoted cell death in a dose dependent manner, reducing the viability of about 40% after 48 h of 2 µM treatment (Figure 10b). This impact was more notable using higher concentration. Contrary, the same concentrations and time exposure in MCF-10A did not trigger evident toxicity and the living cells were recorded to be 85%, 83% and 81% using 2 µM, 2.5 µM and 3 µM of green Ag NPs respectively (Figure 10d). The concentration of 1 µM both at 24 h and 48 h did not cause noticeable alterations in cell viability. The IC50 values, calculated as described in the section Materials, were reported in Table 2, confirming the different toxicity behavior of the two types of Ag NPs in MCF-7 and MCF-10A. In particular, the green Ag NPs presented more effectively than conventional Ag NPs in cancer cell lines reported lower values both at 24 h and 48 h (1.6 µM and 2 µM) compared to the conventional Ag NPs (2 µM and 2.9 µM).

Therefore, the data showed that plant extracts not only were responsible for the reduction and stabilization of cells but also acting as therapeutic agents [92]. The combined action between the green compounds (polyphenols, proteins, and others) and Ag NPs showed a synergistic effect against cancer cell lines [80]. On the other hand, the biomolecules protected the healthy and immune cells from the toxicity and inflammatory properties correlated to the Ag NPs [93].

These results were very significant for future applications of green NPs in animal models.

## 4. Conclusions

In this work, we synthesized Ag NPs using two different techniques, one conventional and one using plant extracts. After the evaluation of their physicochemical properties, we tested their ability to induce an eventual inflammatory response in macrophages. This is particularly important since the inflammation process appears to be highly limiting in terms of using these nanostructures in vivo. Surprisingly, the Ag NPs obtained by green chemistry were well tolerated by macrophages, which do not appear to be activated. This was demonstrated by evaluating the NF-κB translocation, the activation of the cytokines IL-6, IL-8 and TNF-α, the expression of COX-2 and the morphological alterations. On the contrary, the NPs obtained by the conventional technique induced a strong inflammatory state in the cells. At this point, we evaluated the ability of green Ag NPs to act as anticancer agents using MCF-7 cells, demonstrating a reduction in viability of about 55% using 2 µM. We also analyzed the possible toxicity on MCF-10A, which represents the healthy counterpart of MCF-7. In these cells, green Ag NPs did not cause cytotoxicity, contrary to the data obtained using conventional NPs. These results demonstrated the advisability of using green NPs in the biomedical field as they did not stimulate an anti-inflammatory response in vitro. Finally, they were toxic exclusively for tumor lines.

## Figures and Tables

**Table 1:** Characterization of conventional and green Ag NPs in water, DMEM, and RPMI by DLS and ζ-potential measurements.

**Samples in Water**	**DLS (nm)**	**Zeta Potential (mV)**
Conventional Ag NPs	20 ± 3	−30 ± 3
Green Ag NPs	32 ± 6	−35 ± 4
**Samples in DMEM**	**DLS (nm)**	**Zeta Potential (mV)**
Conventional Ag NPs	29 ± 2	−38 ± 2
Green Ag NPs	36 ± 4	−41 ± 3
**Samples in RPMI-1640**	**DLS (nm)**	**Zeta Potential (mV)**
Conventional Ag NPs	31 ± 5	−40 ± 5
Green Ag NPs	38 ± 3	−43 ± 2

**Table 2:** IC50 values calculated on data measured by viability assays reported in Figure 10.

**Conventional Ag NPs**	**MCF-7 (24 h)**	**MCF-7 (48 h)**	**MCF-10A (24 h)**	**MCF-10A (48 h)**
IC50	2 µM	2.9 µM	2.05 µM	2.4 µM
**Green Ag NPs**	**MCF-7 (24 h)**	**MCF-7 (48 h)**	**MCF-10A (24 h)**	**MCF-10A (48 h)**
IC50	1.6 µM	2 µM	1.3 µM	1.4 µM

## Data Availability

The data presented in this study are available in this article.
